# Nomogram predicted risk of peripherally inserted central catheter related thrombosis

**DOI:** 10.1038/s41598-017-06609-x

**Published:** 2017-07-24

**Authors:** Nan Hao, Xin Xie, Zhangjian Zhou, Jieqiong Li, Li Kang, Huili Wu, Pingli Guo, Chengxue Dang, Hao Zhang

**Affiliations:** 1 Department of Surgical Oncology, The First Affiliated Hospital of Xi’an Jiaotong University, 227W Yanta Road, Xi’an, 710061 Shaanxi China; 2 Department of Nurse, The First Affiliated Hospital of Xi’an Jiaotong University, 227W Yanta Road, Xi’an, 710061 Shaanxi China; 3 Department of Thoracic Surgery Ward 2, The First Affiliated Hospital of Xi’an Jiaotong University, 227W Yanta Road, Xi’an, 710061 Shaanxi China; 4 Department of Oncology, The First Affiliated Hospital of Xi’an Jiaotong University, 227W Yanta Road, Xi’an, 710061 Shaanxi China; 5 Department of Breast Surgery, The First Affiliated Hospital of Xi’an Jiaotong University, 227W Yanta Road, Xi’an, 710061 Shaanxi China

## Abstract

The use of peripherally inserted central catheters (PICCs) is increasing rapidly worldwide. A number of patient-related, clinical-related and device-related characteristics might be risk factors for PICC-related thrombosis. We retrospectively reviewed a database of 320 consecutive patients who underwent PICC insertion between December 2014 and December 2015 at the First Affiliated Hospital of Xi’an Jiaotong University to explore the potential associations between risk factors and PICC-associated thrombosis. A novel nomogram for predicting risk was developed based on the data. The nomogram prediction model included ten risk factors that were derived from different relevant estimates. The nomogram prediction model showed good discriminatory power (Harrell’s C-index, 0.709) and a high degree of similarity to actual thrombosis occurring after calibration. Furthermore, principal component analysis was performed to identify the factors that most influence PICC-related thrombosis. Our novel nomogram thrombosis risk prediction model was accurate in predicting PICC-related thrombosis. Karnofsky performance scores, D-dimer and blood platelet levels and previous chemotherapy were principal components. Our findings might help clinicians predict thrombosis risk in individual patients, select proper therapeutic strategies and optimize the timing of anticoagulation therapy.

## Introduction

The use of peripherally inserted central catheters (PICCs) is increasing rapidly worldwide^1, 2^. PICCs are a relatively safe and cost-effective method to provide long-term intravenous access for extended antibiotic injection, chemotherapy, and total parenteral nutrition (TPN)^3^. Chemotherapeutic agents, such as docetaxel, vinorelbine, epirubicin and others, induce extremely strong vascular stimulation. For patients who need chemotherapy, central venous catheters (CVCs), PICCs or implanted transfusion ports are needed to reduce the risks of the potential chemotherapeutic agent-related vascular complications. Considering their safety and cost-effectiveness, PICCs are widely used for patients with cancer who need chemotherapy in China. And they are easy to insert, and associated to a low rate of early and late complications compared to conventional percutaneous method. Furthermore, PICCs are also easy to remove and less expensive than some other devices^4, 5^. However, PICCs have their own risks, such as infection, phlebitis and thrombosis^6^. In recent years, ultrasound technology combined with the Seldinger technique for PICCs has been found to increase the chance of success of PICC puncture and decrease the chance of complications^7^. However, both patients and doctors are concerned with PICC-related thrombosis. A number of patient-related, clinical-related and device-related characteristics might be potential risk factors for PICC-related thrombosis^8^.

The nomogram is a statistics-based tool that provides the overall probability of a specific outcome. This approach can assign every risk factor a single risk score, and a combination of several risk factors can provide a potential incidence rate of the specific endpoint. Nomograms have been widely used to estimate the overall survival rate for many cancers. Furthermore, for many cancers, the nomogram has been demonstrated to be more accurate in predicting individualized survival than the traditional malignant tumour staging systems^9^. This approach can serve as a model for estimating the risk of a clinical outcome.

The objective of this study was to determine whether the use of long-term PICCs was associated with an increased risk of thrombosis. We also identified the observed factors that would place patients at a high risk for thrombosis. We hypothesized that the presence of a long-term PICC would increase the thrombosis risk compared with not having one. In addition, we aimed to develop a nomogram estimation system that could provide clinicians a reference to help determine who might be at high risk for PICC-related thrombosis.

## Results

Between December 2014 and December 2015, 432 PICCs were inserted at our academic central venous access centre. Of these, 81 were removed without a documented removal date, 11 did not have the exact required data and 20 were in patients less than 18 years old. Therefore, our final study cohort included 320 PICCs, accounting for a total of 44,201 catheter days.

The average age of our patients who underwent PICC insertion was 55.85 ± 13.95 years with a range of 18–93 years. Males and females had nearly the same rate of insertion. Most patients underwent insertion via the basilic vein. Almost all patients were given single-lumen, non-powered, non-tunnelled PICC tubes. Fifty (15.6%) of the tube cacumen positions were beyond the sixth thoracic vertebra just after the insertion, 23 of them were at the junction of the superior vena cava and left atrium, and 27 of them were adjusted so that the tip position was in the correct location. Among the 320 patients, 13 (4.1%) had a thrombosis history, 11 (3.4%) had a previous history of PICC insertion, and 34 (10.6%) had received chemotherapy via the peripheral vein before the PICC insertion. Two hundred and thirty-nine patients were treated with active chemotherapy at the time of hospitalization and were diagnosed as having a malignant tumour. The remaining patients were receiving total parenteral nutrition or long-term antibiotic administration. The Karnofsky performance scores (KPS), white blood cell (WBC) count, blood platelet (PLT), fibrinogen concentration (FIB), fibrinogen degradation product (FDP) level, D-dimer and other details are shown in Table 1.

**Table 1:** The univariate analysis of the PICC related thrombosis.

	Entire cohort	No thrombosis	Thrombosis	P value
	n = 320	n = 234	n = 86	
Age	55.85 ± 13.95 (18–93)	56.00 ± 13.20 (18–89)	55.43 ± 15.89 (18–93)	0.878
Gender				
Male	159 (49.7%)	112 (47.9%)	47 (54.7%)	
Femal	161 (50.3%)	122 (52.1%)	39 (45.3%)	0.314
BMI	22.69 ± 7.55 (14.38–36.05)	22.38 ± 3.25 (14.38–36.05)	22.18 ± 2.88 (15.92–29.38)	0.493
Insertion vein				
Basilic	237 (74.1%)	171 (73.1%)	66 (76.7%)	
Brachial	27 (8.1%)	28 (12.0%)	9 (10.5%)	
Cephalic	23 (7.2%)	18 (7.7%)	5 (5.8%)	
Median	33 (10.6%)	17 (7.3%)	6 (7.0%)	0.442
Insertion arm				
Left	165 (51.6%)	124 (53.0%)	41 (47.7%)	
Right	155 (48.4%)	110 (47.0%)	45 (52.3%)	0.450
PICC/Biceps circumference index^a^	9.94 ± 1.28 (7.18–17.94)	9.93 ± 1.32 (7.18–17.94)	9.98 ± 1.16 (8.10 ± 15.70)	0.455
Insertion attempts	1.14 ± 0.42 (1–3)	1.14 ± 0.42 (1–3)	1.14 ± 0.44 (1–3)	0.814
Lumens				
One	318 (99.4%)	234 (100.0%)	84 (97.7%)	
Two	2 (0.6%)	0 (0.0%)	2 (2.3%)	0.072
Power-PICC				
Yes	4 (1.3%)	2 (0.9%)	2 (2.3%)	
No	316 (98.8%)	232 (99.1%)	84 (97.7%)	0.293
Tunneled-PICC				
Yes	3 (0.9%)	1 (0.4%)	2 (2.3%)	
No	317 (99.1%)	233 (99.6%)	84 (97.7%)	0.177
French (gauge)				
4 F	318 (99.4%)	234 (100.0%)	84 (97.7%)	
5 F	2 (0.6%)	0 (0.0%)	2 (2.3%)	0.072
Cacumen position^b^				
Upper than 6	50 (15.6%)	33 (14.1%)	17 (19.8%)	
Lower than 6	270 (84.4%)	201 (85.9%)	69 (80.2%)	0.227
PICC length (cm)	45.61 ± 4.43 (36–56)	45.69 ± 4.62 (36–56)	45.38 ± 3.85 (36–55)	0.763
Thrombosis history				
Yes	13 (4.1%)	6 (2.6%)	7 (8.1%)	
No	307 (95.9%)	228 (97.4%)	79 (91.9%)	0.048
PICC history				
Yes	11 (3.4%)	8 (3.4%)	3 (3.5%)	
No	309 (96.6%)	226 (96.6%)	83 (96.5%)	1.000
Chemotherapy history				
Yes	34 (10.6%)	19 (8.1%)	15 (17.4%)	
No	286 (89.4%)	215 (91.9%)	71 (82.6%)	0.023
Nursing care				
Center	155 (48.4%)	105 (44.9%)	50 (58.1%)	
Non-center	165 (51.6%)	129 (55.1%)	36 (41.9%)	0.043
Puncture methods				
Non-sonograpy	143 (44.7%)	109 (46.6%)	34 (39.5%)	
Sonograpy	160 (50.0%)	115 (49.1%)	45 (52.3%)	
Seldinger	17 (5.3)	10 (4.3%)	7 (8.1%)	0.273
Nurse seniority				
Senior	177 (55.3%)	131 (56.0%)	46 (53.5%)	
Junior	143 (44.7%)	103 (44.0%)	40 (46.5%)	0.705
Therapy				
Chemotherapy	239 (74.7%)	178 (76.1%)	61 (70.9%)	
TPN	75 (23.4%)	52 (22.2%)	23 (26.7%)	
Others	6 (1.9%)	4 (1.7%)	2 (2.3%)	0.638
Diagnosis				
Malignant tumor	286 (89.4%)	214 (91.5%)	72 (83.7%)	
Others	34 (10.6%)	20 (8.5%)	14 (16.3%)	0.047
Other complication				
Yes	59 (18.4%)	35 (15.0%)	24 (27.9%)	
No	261 (81.6%)	199 (85.0%)	62 (72.1%)	0.014
KPS	85.73 ± 28.06 (10–100)	88.14 ± 25.76 (10–100)	79.19 ± 32.83 (10–100)	< 0.001
WBC	6.87 ± 3.70 (1.13–35.57)	6.87 ± 3.44 (1.13–30.88)	6.87 ± 4.36 (1.35–35.57)	0.597
PLT	221.48 ± 93.51 (1.03–580.00)	220.46 ± 98.01 (1.03–580.00)	224.26 ± 80.44 (34.00–541.00)	0.831
FIB	1.13 ± 0.55 (0.09–8.80)	1.45 ± 0.63 (0.09–8.80)	1.10 ± 0.24 (0.76–2.92)	0.662
FDP	3.47 ± 1.59 (0.78–19.80)	3.40 ± 1.35 (0.78–8.99)	3.64 ± 2.12 (1.28–19.80)	0.338
D-dimer	2.37 ± 4.98 (0.10–50.00)	1.82 ± 3.61 (0.20–37.60)	3.90 ± 7.37 (0.10–50.00)	0.004
Duration of PICC use (or time to thrombosis)	NA	157.31 ± 68.73 (23–379)	85.93 ± 71.92 (4–390)	

^a^PICC/Biceps circumference index means the diameter of PICC tube/the biceps circumference*100.

^b^The order of thoracic vertebra.

**Figure 1:**
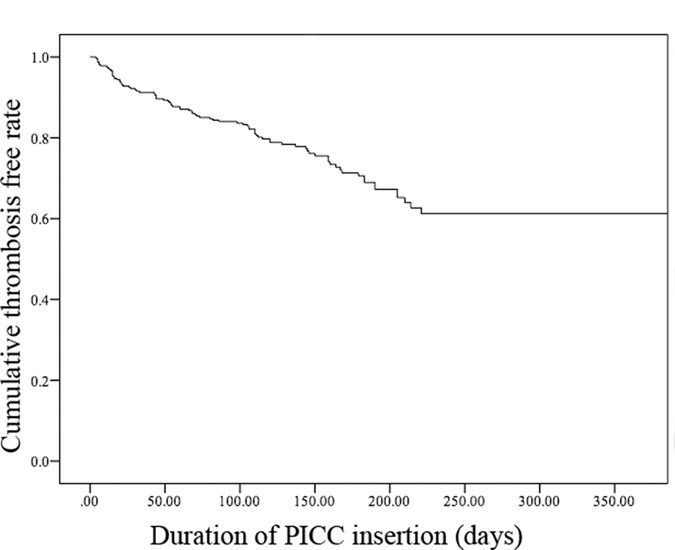
Cumulative thrombosis free rate of the duration of PICC insertion.

Among the 320 included PICCs, 86 (26.9%) had PICC-related thrombosis events that were diagnosed by sonography, 0.19 per 1000 catheter days (Table 1). The mean time to PICC-associated thrombosis was 85.93 days with a range of 4–390 days. The cumulative thrombosis free time of PICC insertion is shown in Fig. 1. Most patients were receiving chemotherapy for a malignant tumour, and 8.1% of the PICC-associated thrombosis patients had a thrombosis history before insertion. Of these patients, 17.4% received chemotherapy before the PICC insertion. As in other reports on the association between thrombosis and infection^10^, almost one-third of our patients with PICC-associated thrombosis experienced preceding complications, such as central line-associated bloodstream infection and phlebitis. With respect to the location of thrombosis, PICC-associated thrombosis more commonly involved the axillary and subclavian veins.

**Figure 2:**
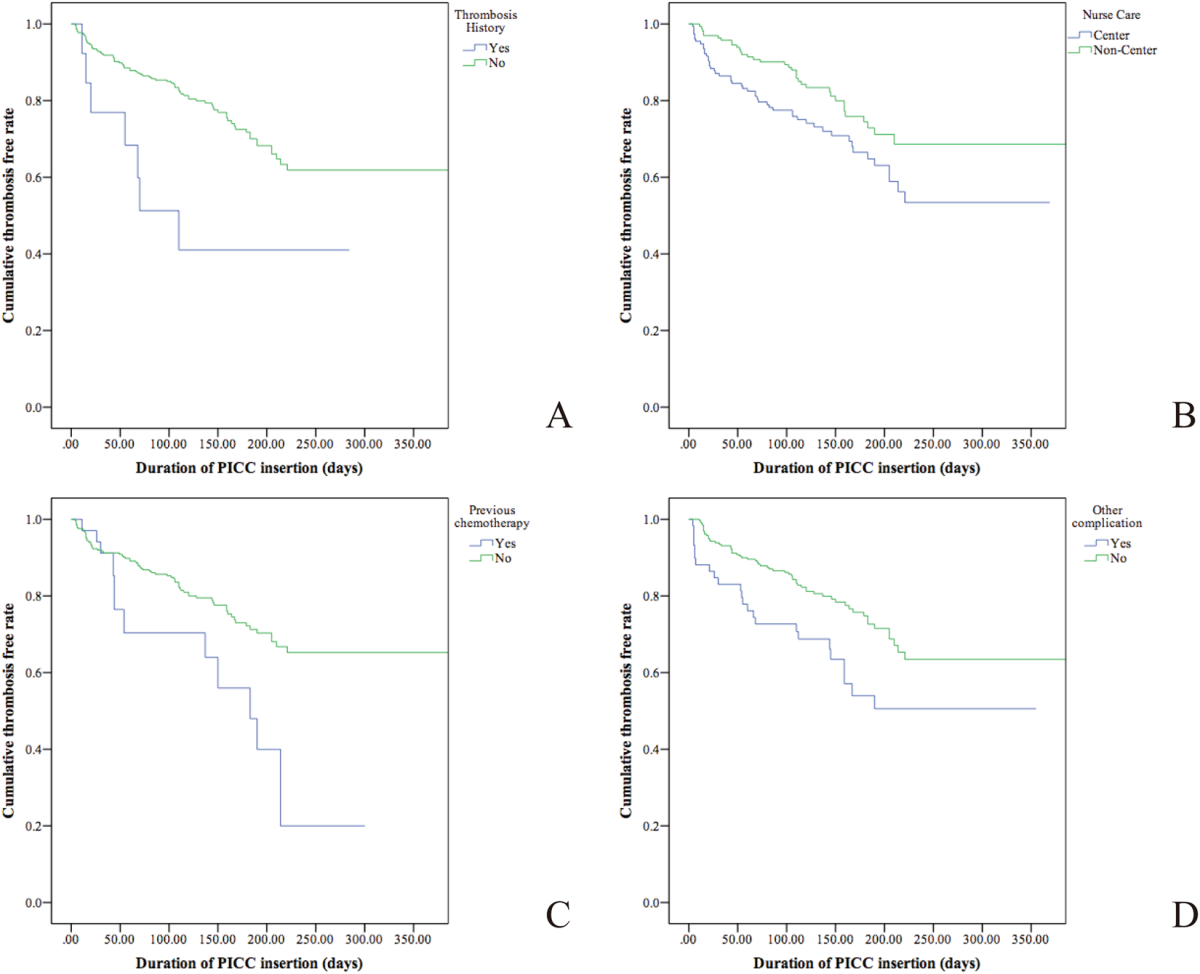
Cumulative thrombosis free rate of the duration of PICC insertion for the dichotomous variables. (**A**) Thrombosis history or not. (**B**) Center care or not. (**C)** Previous chemotherapy or not. (**D**) Have other complications or not.

Seven different risk factors were found to have significant differences in the univariate analysis (Table 1). Previous thrombosis history, receipt of chemotherapy before PICC insertion, subsequent PICC care centre, malignant tumour, other combined complications, KPS scores and D-dimer were risk factors (P < 0.05). According to a bivariate analysis, the cumulative rate of thrombosis free time of the dichotomous variables is shown in Fig. 2.

In our multivariable logistic regression test, the value of the D-dimer test, previous KPS score estimation, other complications during the period of PICC insertion and care centre of the catheter remained associated with PICC-related thrombosis (Table 2).

**Table 2:** The multivariable logistic regression test of the risk factors of PICC related thrombosis.

	Chi-square value	P value
Thrombosis history	2.254	0.133
Chemotherapy history	0.413	0.521
Nursing care	4.173	0.041
Diagnosis	0.887	0.346
Other complication	6.288	0.012
KPS	35.501	0.003
D-dimer	130.605	0.03

For patients who underwent PICC insertion, seven risk factors were included in the nomogram model, including previous thrombosis history, chemotherapy before PICC insertion, subsequent PICC care centre, malignant tumour, other combined complications, KPS scores and D-dimer level, as well as another three thrombosis risk factors that were in the Caprini thrombosis risk assessment guide^11^, namely, the age at the time of PICC insertion, BMI index and blood platelet count. In the nomogram model (Supplement 1), each factor from the multivariate logistic regression model was ascribed a weighted point that implied the risk of thrombosis and the total points were obtained. For each patient, a higher score was considered to predict a higher risk of thrombosis. The predictive accuracies of the nomogram model were evaluated using Harrell’s C-index and the AIC index. For the nomogram model, the C-index was 0.709 with a 338.6564 on the AIC index, which indicated that the model had good discriminatory ability. This result indicated that the nomogram model was good at predicting the risk of PICC-associated thrombosis.

**Figure 3:**
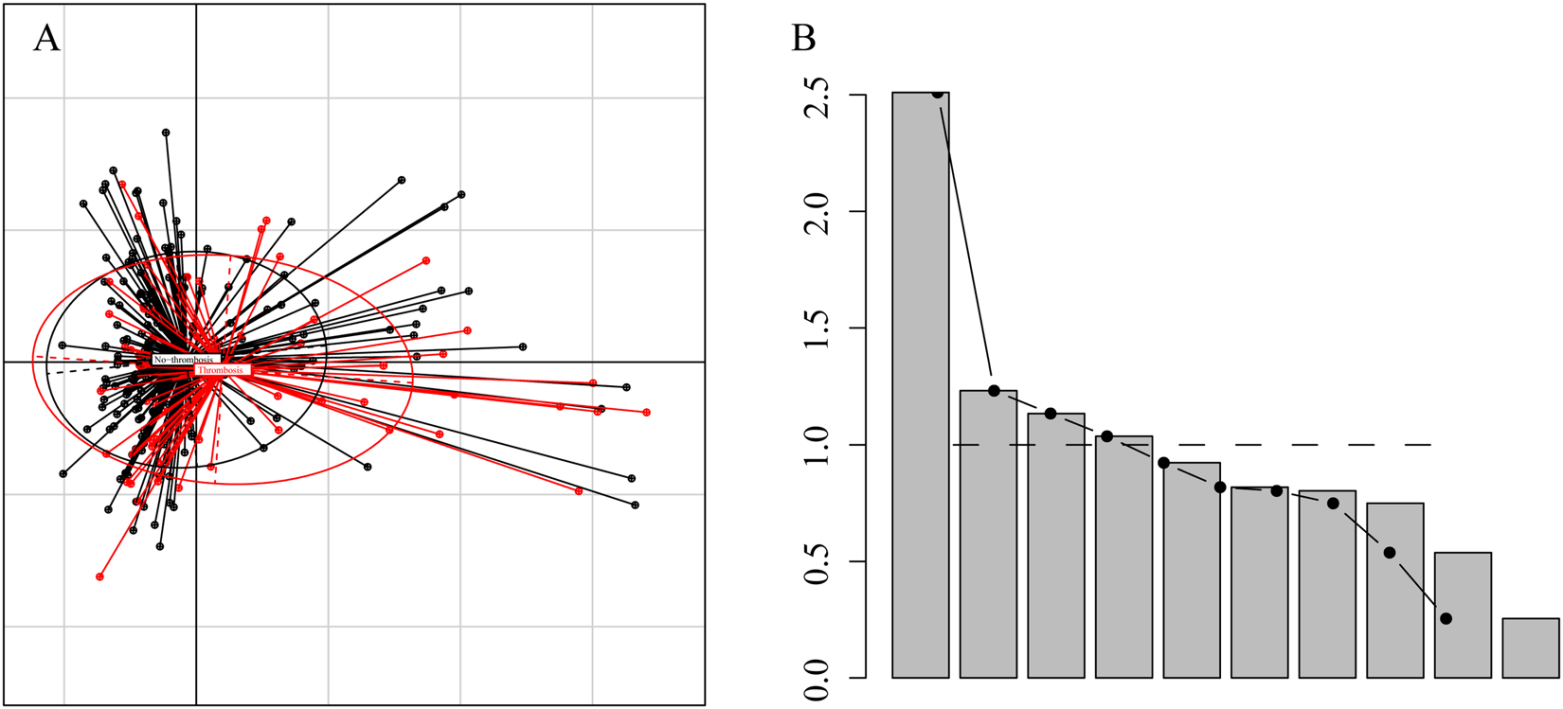
(**A**) The cluster of potential ten risk factors that associated with thrombosis. The plot shown the factorial maps with representation of observation scores. (**B**) The scree plot indicated principal components.

Many clinical factors and device factors were associated with the risk of thrombosis. However, these factors might have a potential relationship with each other; for example, patients who suffered catheter-related complications were more likely to choose a PICC centre with nursing care than home-based care. We performed principal component analysis to identify the factors that were the principal components associated with thrombosis. Figure 3 shows the cluster of ten potential risk factors that are associated with thrombosis, and the scree plot indicated the 4 principal components contributed substantially. Table 3 shows that the 4 principal components were the KPS scores, D-dimer value, blood platelet level and previous chemotherapy; these factors might play an important role in PICC-associated thrombosis formation.

**Table 3:** The feature vector relative rotation matrix.

	Component 1	Component 2	Component 3	Component 4
Thrombosis history	0.375772485	−0.641236632	0.057221876	−0.054494695
D-dimer	0.002995992	0.757616229	−0.006542897	−0.032237601
KPS	0.76263815	−0.33792072	−0.19576948	−0.045470598
Chemotherapy history	−0.081525343	−0.138597556	0.090880607	0.751294508
Other complication	0.081440799	−0.177518729	0.673307864	−0.013228507
Diagnosis	−0.749126013	0.369199151	0.195301788	0.104600104
Age	−0.310621949	0.193010276	0.334725716	0.310324949
PLT	0.160434684	−0.126044023	−0.738375364	0.074869299
Care center	0.728618386	0.326886316	0.215231555	0.053229872
BMI	−0.042958488	−0.1258256	0.181293843	−0.731595534

**Figure 4:**
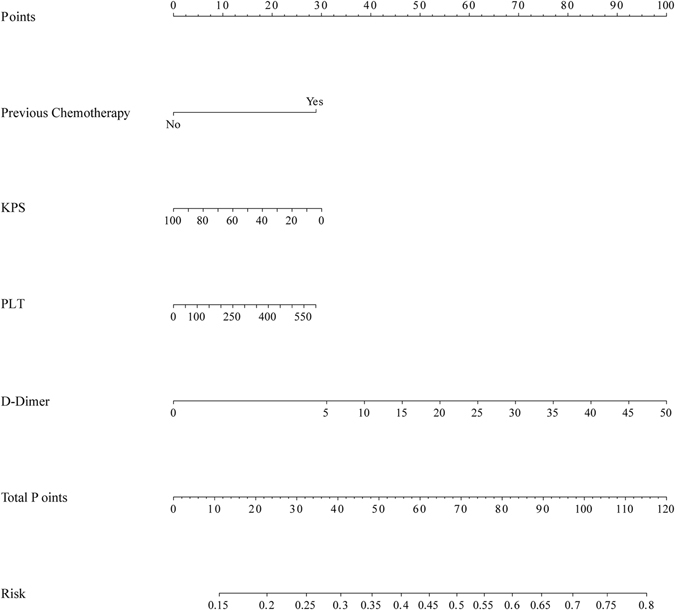
Nomogram predicted PICC associated thrombosis risk using the four chief characteristics.

**Figure 5:**
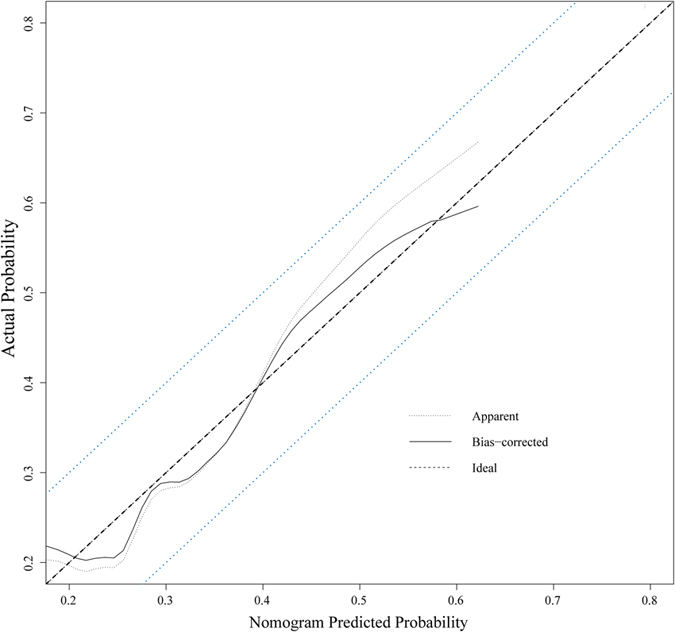
Calibration of the nomogram predicted system. Nomogram predicted probability of thrombosis was plotted on the x-axis, actual PICC associated thrombosis was plotted on the y-axis and 95% CIs measured by logistic regression analysis. All predictions lie within the 10% margin of error (within the blue dots line).

In order to make a user-friendly model for clinicians, the four chief factors were brought into the final nomogram model. The final nomogram model used to predict the risk of thrombosis for patients with PICC insertion is shown in Fig. 4 with a C-index of 0.641. Further, Fig. 5 shows the calibration plot of the risk of thrombosis. As indicated in this figure, the predicted risk of the PICC-associated thrombosis corresponded closely with actual thrombosis occurrence and was within a 10% margin of error. Further, another external dataset fulfilled the inclusion criteria was used to validate the nomogram model, and reach a C-index of 0.769 (Supplement 2). The details of the validation set were shown in Supplement 3. In order avoid the bias due to the anti-thrombosis therapy, the patients received preventive anti-thrombosis therapy were excluded from the validation set and the rest reached a C-index of 0.705 (the calibration curve was shown in Supplement 4)

## Discussion

Peripherally inserted central catheters are used widely and increasingly in patients with tumours. Compared with centrally inserted venous catheters or vein-detained needles, PICC lines have several advantages: they are convenient and easy to operate, stable and safe to insert, and carry a low risk of vascular injury and blood infections for patients with longer PICC application^3^. However, catheter-related deep vein thrombosis is a common complication. Furthermore, thrombosis can be asymptomatic and can lead to a potentially serious complication, such as pulmonary embolism, which is an acute and lethal syndrome for patients with tumours^12^. Therefore, the estimation and management of PICC utilization are critical for patient health and prognosis.

PICC-related thrombosis consists of blood clots in veins or catheter insertion-associated damage of the vascular intima^13^. Based on the theory of Virchow’s triad, the formation of a thrombus requires three components: abnormal blood flow, vascular endothelial damage and hypercoagulable blood^14^. To investigate the potential risk factors for PICC-related thrombosis, these three critical components should be preferentially explored.

According to Virchow’s triad, the PICC device itself may increase the chances of thrombosis. Based on an experimental model of catheter-related thrombosis, Nifong *et al*. demonstrated that the insertion of a PICC resulted in a decrease in laminar blood flow with an increase in turbulent flow because of the obstruction of the PICC device, which may influence the stability of hydrodynamics and normal blood flow^15^. Meanwhile, the mechanical injury of the vascular wall caused by PICC insertion could activate platelets and the clotting system, leading to local vasoconstriction, platelet adhesion and fibrin generation. These processes will eventually induce thrombosis.

For patients with malignant tumours, chemotherapy is one of the most important and effective therapies and can improve the patients’ prognosis. As chemotherapy regimens can cause serious damage to the vascular intima and induce phlebitis, central venous instead of peripheral venous catheters are chosen for regimen infusion, including PICCs. Although the local concentration of chemotherapy regimens could be diluted by the rapid blood flow in a central venous catheter, it will also cause vascular endothelial damage and local inflammation because of the infusion of chemo-agents. Therefore, chemotherapy is generally accepted as a risk factor for thrombosis. Lyman *et al*. showed that the risk of thrombosis increased by more than 6-fold for patients with malignant tumours and a history of chemotherapy^16^. Chronic inflammation induced by chemotherapy agent infusion could cause vasoconstriction and change the haemodynamics, facilitating the occurrence of thrombosis. Furthermore, chemotherapy regimens may alter the local pH of blood, which can directly affect the venous endothelium and promote thrombus formation.

In recent studies, many factors have been identified as risk factors of vascular thrombosis, such as the number of lumens of the PICC device, side of line placement, catheter tip location, prior history of thrombosis, surgery lasting more than 1 hour, diabetes and malignancy^17, 18^. These risk factors can help predict and estimate the status of PICC-related thrombosis, enabling the accurate prediction and early prevention of thrombus-associated complications. In our study, we found that the D-dimer, platelet count and KPS could also be accurate risk factors.

In modern oncology practice, KPS is commonly used to evaluate the patients’ performance status, which includes personal performance, activity ability, self-care ability and so on^19^. According to KPS, cancer patients with a KPS more than 60 are candidates to receive standard anti-cancer therapies. For cancer patients who are given a PICC, the KPS remains suitable to assess the entire status of patients. In our study, we found that KPS could be a potential risk factor for PICC-related thrombosis. A lower KPS and pre-operative deficit may make it more difficult for these patients to ambulate, thus predisposing them to developing thrombosis^20, 21^. D-dimer is a degradation product of fibrin during fibrinolysis; therefore, it is considered a predictor for both fibrinolysis and coagulability. In several studies, D-dimer was demonstrated to be an effective predictor of prognosis in solid tumours^22, 23^. Meanwhile, as for patients with malignant tumours, D-dimer raises the risk of vascular thrombosis by approximately 4-fold^24^. Guido *et al*. showed that D-dimer could predict venous thromboembolism before chemotherapy for patients with malignant tumours^25^. In our study, we found that D-dimer, as a non-linear factor, increases the risk of PICC-associated thrombosis as its value increases. Therefore, we consider D-dimer as an accurate and low-cost risk factor for PICC-related thrombosis.

We developed a novel thrombosis prediction system for patients with PICC insertion, namely, a nomogram prediction model. Based on the cross-sectional analysis of data from 320 patients who underwent PICC insertion, we utilized four factors to predict thrombosis in patients with PICC insertion in the nomogram model. Each factor included in the nomogram model was ascribed a weighted point to estimate the effect of this factor on thrombosis prognosis. In the nomogram model, a higher score indicated a higher risk of thrombosis formation. Then, we appraised the predictive accuracy and homogeneity of the model by calculating Harrell’s C-index and the AIC index, and the results suggested that the nomogram prediction model corresponded closely with actual thrombosis occurrence, with a C-index greater than 0.7, indicating that the nomogram model was an effective prediction system^26, 27^. In our nomogram model, the location at which patients received PICC care was a critical factor associated with the risk of thrombosis. However, patients who received catheter nursing in our centre have a higher risk of thrombosis than those who received only home-based catheter care. After sub-group analysis, we found that patients who suffered complications or serious diseases were more likely to choose a central centre for catheter care. Further, principal component analysis revealed a series of significant factors accounting for the variation between patients with and without thrombosis. The score outputs demonstrated that the KPS scores, D-dimer value, blood platelet level and previous chemotherapy are the four principal component coordinates. This result is consistent with the results of our nomogram model.

In conclusion, our novel nomogram thrombosis risk prediction model, which included four clinical factors, generated estimates that accurately predicted PICC-related thrombosis. Among these, KPS scores, D-dimer level, PLT and previous chemotherapy were principal components. These findings might help clinicians predict the thrombosis risk in individual patients, select proper therapeutic strategies and optimize the timing of anticoagulation therapy.

## Materials and Methods

### Patients

In this cross-section study, we retrospectively reviewed a database of 320 consecutive patients who underwent PICC insertion between December 2014 and December 2015 at the First Affiliated Hospital of Xi’an Jiaotong University. Demographic and clinical information, including the age, sex, indication for insertion, number of insertion attempts, vein and arm of insertion, and details regarding the type of PICC, was included in our study. This investigation was approved by the ethical committee of the First Affiliated Hospital of Xi’an Jiaotong University. Written informed consent was obtained from all patients for participation in this study and inclusion of their data and any accompanying images in this report.

The inclusion criteria were as follows: 1. Patients who required PICC insertion as a clinical requirement; 2. available demographic information and clinical information; 3. patients who survived for more than three months after insertion; 4. patients who were more than 18 years old, and 5. patients who did not have a contraindication for PICC insertion.

All PICC insertions were performed according the protocols of the peripherally inserted central catheter nursing group at our hospital. The position of the PICC tips was verified by chest X-ray. PICC-associated thrombosis was verified using B-mode Doppler ultrasound.

### Statistical analysis

Continuous data are presented as the means ± standard deviations. Categorical variables were grouped and compared using the χ2 test or Fisher’s exact test. Continuous variables were compared using Student’s t-test. Univariate and multivariate logistics regression models were constructed to explore the associations between clinical factors and the risk of thrombosis. Principal component analysis is a classic dimension reduction approach which has been shown to have better prediction performance and interpretability in data analysis. After principal component analysis, preselected multiple potential interactions were tested as nomogram parameters irrespective of significance^28, 29^. 200 bootstrap re-sample method was used to make the internal validation strategy^30, 31^. Akaike’s Information Criterion (AIC) and Harrell’s C statistic were used to estimate the accuracies and relative discriminatory abilities of the predictions. All statistical tests were two-sided, and P values < 0.05 were considered statistically significant. Statistical analyses were performed using SPSS 17.0 (Chicago, IL, USA) and R software version 3.3.0 (http://www.r-project.org) with the “rms”, “ade4” and “AICcmodavg” packages.

## Electronic supplementary material

Supplementary Information
