# Supplementary material for: Nomogram predicted risk of peripherally inserted central catheter related thrombosis
Source: Sci Rep. 2017 Jul 24;7:6344. doi: 10.1038/s41598-017-06609-x (PMC5524883; doi:10.1038/s41598-017-06609-x)

Nomogram predicted risk of peripherally inserted central catheter related thrombosis

Nan Hao[1], Xin Xie[1], Zhangjian Zhou[1], Jieqiong Li[2], Li Kang[3], Huili Wu[4], Pingli Guo[5], Chengxue Dang[1], Hao Zhang[1]

1 Department of Surgical Oncology, The First Affiliated Hospital of Xi'an Jiaotong University, 227W Yanta Road, Xi'an, 710061, Shaanxi, China.

2 Department of Nurse, The First Affiliated Hospital of Xi'an Jiaotong University, 227W Yanta Road, Xi'an, 710061, Shaanxi, China.

3 Department of Thoracic Surgery Ward 2, The First Affiliated Hospital of Xi'an Jiaotong University, 227W Yanta Road, Xi'an, 710061, Shaanxi, China.

4 Department of Oncology, The First Affiliated Hospital of Xi'an Jiaotong University, 227W Yanta Road, Xi'an, 710061, Shaanxi, China.

5 Department of Breast Surgery, The First Affiliated Hospital of Xi'an Jiaotong University, 227W Yanta Road, Xi'an, 710061, Shaanxi, China.

Supplement 1

Seven risk factors were included in the primary nomogram model (shown below), including previous thrombosis history, chemotherapy before PICC insertion, subsequent PICC care centre, malignant tumour, other combined complications, KPS scores and D-dimer level, as well as another three thrombosis risk factors that were in the Caprini thrombosis risk assessment guide, namely, the age at the time of PICC insertion, BMI index and blood platelet count.

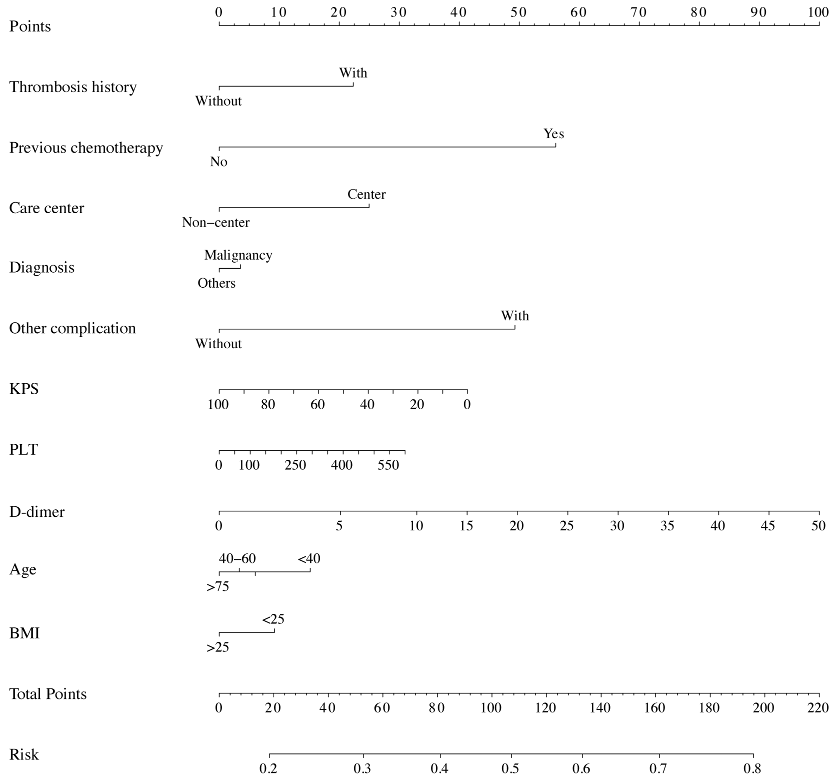

Supplement 2

Another external dataset fulfilled the inclusion criteria was used to validate the nomogram model, and reach a C-index of 0.769. The calibration cure the external validation set was shown below. The calibration cure of the external validation set. Nomogram predicted probability of thrombosis was plotted on the x-axis, actual PICC associated thrombosis was plotted on the y-axis and 95 % CIs measured by logistic regression analysis. 10 % margin of error was within the blue dots line.

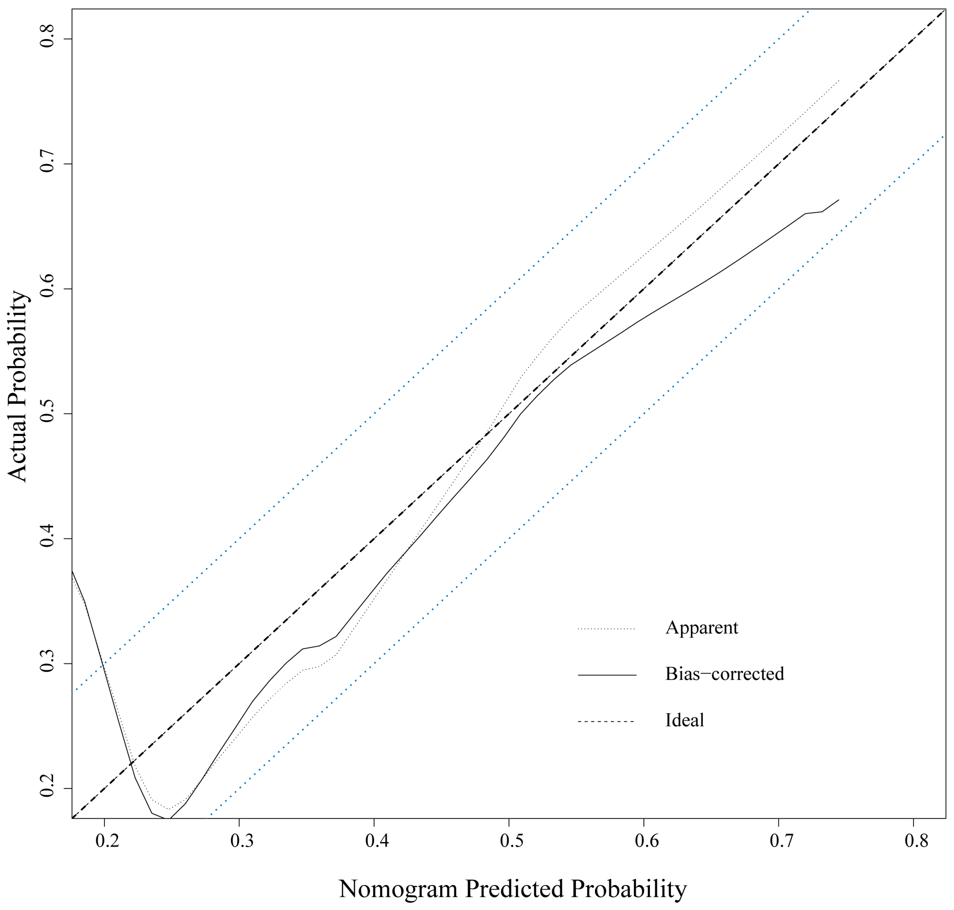

Supplement 3 The univariate analysis of the PICC related thrombosis in the validation set

	Entire cohort<br>n = 120	No thrombosis<br>n = 88	Thrombosis<br>n = 32	P value
Age	57.38±12.65 (22-89)	58.78±11.52 (27-89)	53.53±14.87 (22-82)	0.090
Gender				
Male	68 (56.7%)	49 (55.7%)	19 (59.4%)	
Femal	52 (43.3%)	39 (44.3%)	13 (40.6%)	0.718
BMI	21.55±2.96 (14.49-29.37)	21.71±2.97 (14.49-29.37)	21.14±2.91 (15.91-27.45)	0.305
Insertion vein				
Basilic	97 (80.8%)	70 (79.5%)	27 (84.4%)	
Brachial	5 (4.2%)	4 (4.5%)	1 (3.1%)	
Cephalic	6 (5.0%)	5 (5.7%)	1 (3.1%)	
Median	12 (10.0%)	9 (10.2%)	3 (9.4%)	0.918
Insertion arm				
Left	63 (52.5%)	48 (54.5%)	15 (46.9%)	
Right	57 (47.5%)	40 (45.5%)	17 (53.1%)	0.537
PICC/Biceps circumference index[a]	9.86±1.28 (7.85-17.94)	9.72±1.36 (7.85-17.94)	10.23±1.00 (8.37±12.56)	0.005
Insertion attempts	1.67±0.49 (1-3)	1.17±0.48 (1-3)	1.16±0.51 (1-3)	0.678
Lumens				
One	120 (100.0%)	88 (100.0%)	32 (100.0%)	
Two	0 (0.0%)	0 (0.0%)	0 (0.0%)	NA
Power-PICC				
Yes	0 (0%)	0 (0.0%)	0 (0.0%)	
No	120 (100.0%)	88 (100.0%)	32 (100.0%)	NA

Tunneled-PICC				
Yes	0 (0%)	0 (0.0%)	0 (0.0%)	
No	120 (100.0%)	88 (100.0%)	32 (100.0%)	NA
French (gauge)				
4F	120 (100.0%)	88 (100.0%)	32 (100.0%)	
5F	0 (0%)	0 (0.0%)	0 (0.0%)	NA
Cacumen position[b]				
Upper than 6	9 (7.5%)	4 (4.5%)	5 (15.6%)	
Lower than 6	111 (92.5%)	84 (95.5%)	27 (84.4%)	0.056
PICC length (cm)	45.18±4.16 (36-56)	44.95±4.41 (36-56)	45.81±3.36 (38-52)	0.174
Thrombosis history				
Yes	0 (0%)	0 (0.0%)	0 (0.0%)	
No	120 (100.0%)	88 (100.0%)	32 (100.0%)	NA
PICC history				
Yes	3 (2.5%)	1 (1.1%)	2 (6.3%)	
No	117 (97.5%)	87 (98.9%)	30 (93.8%)	0.173
Chemotherapy history				
Yes	9 (7.5%)	4 (4.5%)	5 (15.6%)	
No	111 (92.5%)	84 (95.5%)	27 (84.4%)	0.056
Nursing care				
Center	58 (48.3%)	41 (46.6%)	17 (53.1%)	
Non-center	62 (51.7%)	47 (53.4%)	15 (46.9%)	0.543
Puncture methods				
Non-sonograpy	40 (33.3%)	31(35.2%)	9 (28.1%)	
Sonograpy	72 (60.0%)	54 (61.4%)	18 (56.3%)	

Seldinger	8 (6.7%)	3 (3.4%)	5 (15.6%)	0.058
Nurse seniority				
Senior	69 (57.5%)	54 (61.4%)	15 (46.9%)	
Junior	51 (42.5%)	34 (38.6%)	17 (53.1%)	0.210
Therapy				
Chemotherapy	93 (77.5%)	68 (77.3%)	25 (78.1%)	
TPN	27 (22.5%)	20 (22.7%)	7 (21.9%)	1.000
Others	0 (0.0%)	0 (0.0%)	0 (0.0%)	
Diagnosis				
Malignant tumor	117 (97.5%)	85 (96.6%)	32 (100.0%)	
Others	3 (2.5%)	3 (3.4%)	0 (0.0%)	0.564
Other complication				
Yes	18 (15.0%)	10 (11.4%)	8 (25.0%)	
No	102 (85.0%)	78 (88.6%)	24 (75.0%)	0.083
KPS	93.13±18.66 (10-100)	94.09±18.23 (10-100)	90.47±19.85 (10-100)	0.013
WBC	6.44±2.52 (2.21-14.76)	6.63±2.42 (1.85-14.76)	5.91±2.74 (2.21-14.36)	0.103
PLT	228.68±85.54 (21.00-454.00)	229.22±87.08 (21.00-454.00)	227.22±82.48 (49.00-406.00)	0.964
FIB	1.05±0.20 (0.09-2.17)	1.04±0.23 (0.09-2.17)	1.07±0.08 (0.91-1.27)	0.220
FDP	3.38±1.21 (1.08-8.99)	3.45±1.27 (1.08-8.99)	3.20±1.01 (1.63-5.54)	0.478
D-dimer	1.94±5.52 (0.20-50.00)	1.12±0.98 (0.20-5.50)	4.19±10.34 (0.20-50.00)	0.320
Duration of PICC use (or time to thrombosis)	NA	192.31±41.53 (31-299)	135.93±50.21 (3-282)	

a. PICC/Biceps circumference index means the diameter of PICC tube/the biceps circumference*100

b. The order of thoracic vertebra

Supplement 4

Patients did not receive anti-thrombosis therapy in the external dataset fulfilled the inclusion criteria was used to validate the nomogram model again, and reach a C-index of 0.705. The calibration cure was shown below.

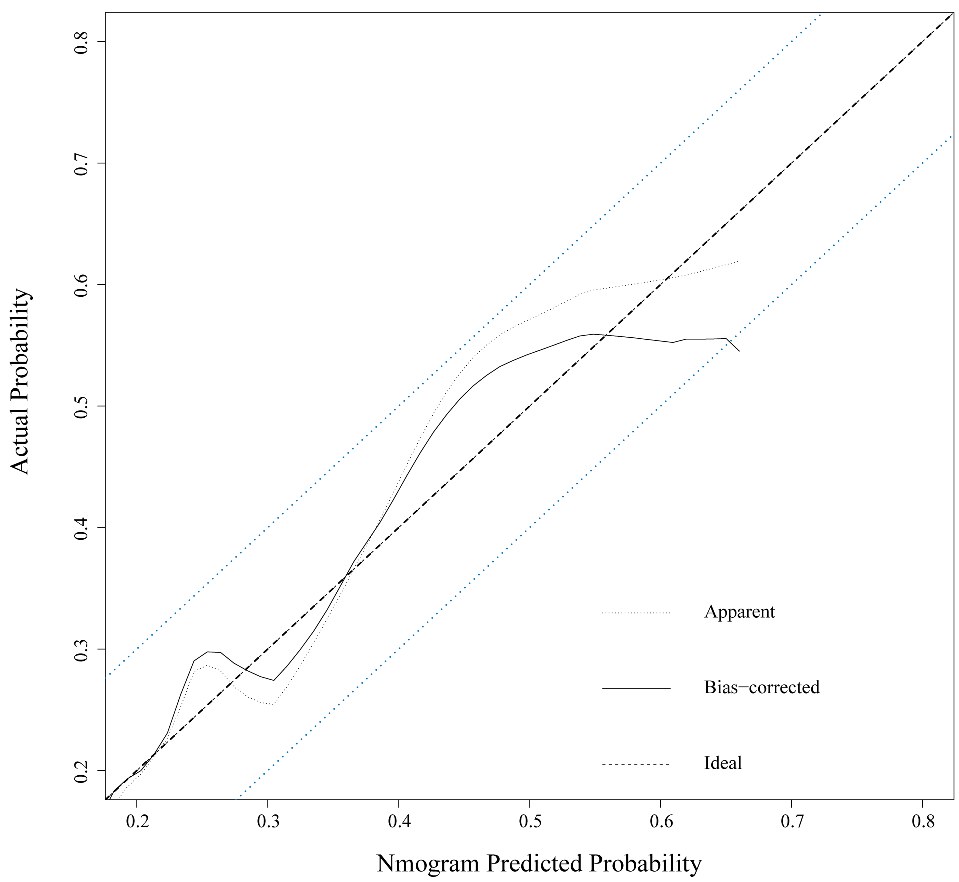

Supplement: Supplementary file 1 — Supplementary Information [file 41598_2017_6609_MOESM1_ESM.pdf]
